# Oral health status and treatment needs among psychiatric inpatients in Rennes, France: a cross-sectional study

**DOI:** 10.1186/1471-244X-13-227

**Published:** 2013-09-21

**Authors:** Valerie Bertaud-Gounot, Viviane Kovess-Masfety, Catherine Perrus, Gilda Trohel, Frederique Richard

**Affiliations:** 1 EHESP School of Public Health, Epidemiology and Biostatistics Department, Avenue Professor Leon Bernard, CS 74312, 35043 Rennes, France; 2 University of Rennes1, Faculty of Dentistry, 2 Avenue du Professeur Léon Bernard (Bât 15), 35043 Rennes cedex, France; 3 University Hospital of Rennes, 2 rue Henri Le Guilloux, 35033 RENNES cedex 9, France; 4 Guillaume Régnier Psychiatric Center, 108, av. du Général Leclerc, BP 60321-35703 Rennes, France

**Keywords:** Dental caries/epidemiology, Dental health survey, Hospital, Psychiatric, Schizophrenia/complications

## Abstract

**Background:**

Severe mental disorders have a chronic course associated with a high risk for co-morbid somatic illnesses and premature mortality and oral health is critical for overall systemic health. But general health care needs in this population are often neglected. Some studies have aimed at determining the oral health status of psychiatric in-patients but to date, no emphasis has been placed on oral health of psychiatric patients in France. The goal of this study was to assess the oral health and treatment needs of institutionalized patients in a large psychiatric hospital, where a dental service was available and free, to compare it with the average population, with psychiatric in-patients in other countries and to provide recommendations for psychiatrists and care-giving staff.

**Methods:**

The dental status (DMFT), the oral hygiene (OHIS: Simplified Oral Hygiene Index), the saliva flow rate were recorded on a randomized patient sample. Demographic and medical data were retrieved from the institutional clinical files.

**Results:**

Among the 161 examined patients, 95 (59.0%) were men and 66 (41.0%) were women. The mean age was 46.9 ± 17.5 years. The majority was diagnosed schizophrenia (36.6%) or mood disorders (21.1%). The mean OHIS was 1.7 ± 1.1. Among the 147 patients who agreed to carry out the salivary examination, the average saliva flow rate was 0.3 g ± 0.3 g/min. Saliva flow under the average rest saliva flow (0.52 mg/min) was found for 80.3% of the patient. The mean DMFT was 15.8 ± 8.8 (D = 3.7 ± 4.4, M = 7.3 ± 9.4, F = 4.7 ± 4.9) and significantly increased with age (p < 0.001) and degree of disability (p = 0.003) (stepwise linear regression). Eighteen patients (11.2%) were edentulous.

**Conclusions:**

The DMFT was similar to low income French population but psychiatric patients had almost 4 times more decayed teeth, slightly less missing teeth and 1.5 times less filled teeth. Oral health appeared to be better than in most other countries. But compared to general population, the still unmet dental and prosthetic needs indicated the major need of enhanced access to dental care and specific preventive programs.

## Background

Severe mental disorders have a chronic course associated with a high risk for co-morbid somatic illnesses and premature mortality, but despite this increased risk, general health care needs in this population are often neglected [1-3].

In this context, oral health is important because it is critical for overall systemic health. Indeed, bad oral health (periodontitis) is a risk factor for poor glycemic control [4] and cardiovascular disease [5] and there is fair evidence of the association of pneumonia with bad oral health [6]. Moreover, pain and discomfort caused by oral diseases can result in eating difficulties leading to poor levels of nutrition. Bad oral health can affect daily quality of life, well-being and self-esteem.

People with severe mental illness commonly exhibit many factors which may contribute to poor oral health: xerostomia caused by psychiatric medication, lack of motivation for self-care and oral hygiene, tobacco consumption. Dental cost, fear and difficulty in accessing healthcare facilities are the most commonly cited barriers to dental care [7,8].

Indeed, numerous international original studies [3,7-14] and one meta-analysis [15] reported that oral health status of mentally ill patients was poor compared to normal population: in literature, DMFT (number of Decayed, Missing due to decay and Filled Teeth) ranged from 14.9 [9] to 26.7 [8] except in Davengere, India where the very low DMFT (0.9 with mean patients’ age 36.7) [3] was allotted by the authors to the water fluoride concentration (1.5-2 ppm). Multivariate analyses, when carried out, showed that many factors might affect the oral health of these specific patients: DMFS (number of Decayed, Missing or Filled Surfaces) or DMFT was correlated with socio-demographic factors (age [3,12-14,16-18], male gender [12]), psychiatric factors (duration of the mental illness [3,19], psychiatric diagnosis [19], diagnostic of schizophrenia compared to mental retardation [16]), physical factor (high Body Mass Index [20]), care factors (type of ward: open, closed, chronic of special ward [18]; infrequent dental visit [17]) and behavioral factors (irregularity of hygiene habits [3], frequent snacking [17]).

Currently decayed teeth were associated with neglected tooth brushing [8] and perceived xerostomia [19].

The number of missing teeth was associated with age [18], elementary level of education [13], low income [13] and length of stay in institution [13].

The care index (ratio of the number of Filled teeth to the DMFT) was associated with education of only elementary school [13], low income [13] and length of stay in institution [13].

In France, the mandatory health insurance partly covers conservative and surgical dental care (70%), prosthetics and orthodontics treatment (30 to 50%). The complementary health insurance companies or additional insurances coverage depends on the subscribed contract. Since 2000, people who do not work enough (200 h work within the last three months) to get access the mandatory health insurance can benefit from the CMU (universal health insurance). For all low level income people, the CMU is free. They also get access to CMUc (complementary CMU if their annual income is below 7 771€) which includes a basket of free dental prosthetic care. In France in 2007, 4 398 063 people were affiliated to the CMUc, that is 6.8% of the population (3.6% for the Ille-et-Vilaine department). People with severe psychiatric illness usually have very low incomes and can be fully covered by the CMUc. Furthermore, from a financial point of view, somatic treatments (including dental care) are free of charge for patients hospitalized in psychiatric hospitals or in the psychiatric ward of general hospitals. From a practical point of view, some psychiatric hospitals have a somatic care ward which includes dental care or dental consultations. But in France, there is no across-the-board formal connection between psychiatric and somatic treatment and the somatic care of patients undergoing psychiatric treatment remains heterogeneous [21].

Some studies have aimed at determining the oral health status of psychiatric in-patients but to date, no emphasis has been placed on oral health of psychiatric patients in France. Furthermore, few studies [3,11,14,22] were carried out in mental health institutions having a dental department.

The goal of this study was to determine the oral health status and treatment needs of institutionalized patients in a large psychiatric hospital, where a dental service was part of the mental health hospital and to study the possible relationships between DMFT and various variables. The results were compared to the average population and to psychiatric in-patients in other countries reported in literature.

## Methods

### Population

A cross-sectional descriptive survey was carried out in the Guillaume Regnier Hospital of Rennes (Centre Hospitalier Guillaume Regnier: CHGR). This hospital serves all the department of Ille-et-Vilaine (925 000 inhabitants) and has a dental service since the early 70s, where patients can be treated free of charge (opened four half-days a week). Regular screenings are carried out, and treatment is arranged if the patient wishes. Entonox (50% N2O/50% O2) can be used if needed.

The population of this study was all the adult inpatients (741 beds, 659 patients in February 2006).

We calculated that at least 144 patients would be needed (for a difference of the mean DMFT between two groups equal to half the standard deviation to be statically significant with α = 0,05 and the statistical power: 1- β = 0,85). A list of all the beds was drawn up. A random sample of approximately 25% of the beds was chosen for examination (185 beds). By the day of examination, if there was no hospitalized patient corresponding to a sampled bed, the bed immediately after on the list was chosen instead. The data were collected between March and June 2006. Subjects with aggressive behavior and lack of cooperation were excluded. A total of 161 patients were examined. All the procedures were part of standard cares as patients have regular oral and salivary examinations.

### Oral examinations

Clinical examinations were conducted in the psychiatric wards by one dental professional with a mirror, a probe and a transillumination lamp and without the use of radiographs. The patient was sitting on a chair. The clinical assessment was recorded according to the WHO criteria [23]: diseases of the oral mucosa, severity of lifetime accumulated caries experience estimated with the DMFT index (number of Decayed, Missing due to decay and Filled Teeth), oral hygiene with the Simplified Oral Hygiene Index (OHI-S) [24]. The OHIS isthe sum of the plaque index and the calculus index. Both plaque index and calculus index are the means of 6 plaque (calculus) scores assessed on 6 tooth surfaces. 0 = No debris (calculus); 1 = Debris (calculus) on less than 1/3 of the tooth surface; 2 = Debris (calculus) covering between 1/3 and 2/3; 3 = Debris(calculus) covering more than 2/3). Additionally, treatment needs and existing and needed prosthesis were recorded.

### Saliva

The whole saliva flow was also assessed with the “swab method” [25] which was chosen because it didn’t need any active cooperation from the patient. Three swabs (dental cotton rolls) were placed in front of the orifices of the major salivary glands excretory ducts (two in the upper vestibules and one under the tongue). They allowed collecting saliva for five minutes, after which they were put back in their hermetic boxes. Each box containing three swabs was weighted before and after the saliva collection. The mean flow rate measured by the swab method has been reported to be 0.5 ± 0.1 g/min [24]. Bacteriological tests CARIO ANALYSE (P.Fabre) were carried out in order to measure the buffering capacity and the number of Lactobacillus and Streptococcus Mutans.

### Oral health questionnaire

Patients were questioned about their tooth brushing habits (never/less than once a day/once a day or more) and smoking habits (No, 1–9 cigarettes/day, 10–20 cig/d, more than 20 cig/d).

### Degree of disability

Medical team was asked about the patient’s level of autonomy. The patient wasconsidered as self-sufficient person if he was able to perform basic self-care activities such as bathing, dressing and feeding. The others were considered as “partially or totally disabled”.

### Medical record

Demographic and medical variables were retrieved from institutional medical records: age, gender, last psychiatric diagnosis (according to the International Classification of Diseases 10th Revision: ICD-10), prescribed drugs at the time of examination, length of hospitalization (time spent in the psychiatric institution).

### Data analysis

The questionnaires were built and filled with EPI-INFO (TM) Version 3.3.2. The mean OHI-S was computed and the scores were classified into three levels: Excellent (0); Good (0.1-1.2); Fair (1.3-3.0); Poor (3.1-6.0) [26].

Analysis of variance (ANOVA) was used to test the differences in the mean scores of oral hygiene and DMFT and its components using SPSS for Windows Statistical Software Package Version 17.0. Chi-square test was used to test the differences between frequencies. Stepwise logistic regression analysis was used to identify DMFT predictors. Predictors’ candidates were age, gender, diagnosis of psychiatric disorder, length of hospitalization, saliva flow and degree of disability. The level of significance was set at 0.05.

### Ethics

The study received approval from the ethic committee of the hospital (committee of protection of the persons). The study was explained and patient’s written informed consent was obtained from the patients (and from their legal guardians for persons under guardianship).

## Results

The population consisted of 659 patients (February, 7th 2006): 391 men (59.3%; 95% Confidence Interval = [55.5%-63.1%]) and 268 women (40.7% [36.9%-44.5%]). The mean age was 47.3 years.

Of the total sample (185 patients), 24 patients were excluded: 14 subjects refused to participate and 10 were unable to cooperate due to their psychiatric disease. Among the 161 examined patients, 95 (59.0%) were men and 66 (41.0%) were women. There were significantly more men than women in the sample. The sample’s sex ratio was reflective of the population’s sex ratio. The ages ranged from 18 to 90 years (mean age 46.9 ± 17.5) (Table 1).

**Table 1:** Age and gender distribution of the sample

	**Men**		**Women**		**Total**	
**Age (years)**	***n***	***%***	***n***	***%***	***n***	***%***
18 -24	9	9.5	10	15.2	19	11.8
25-34	19	20.0	6	9.1	25	15.5
35-44	21	22.1	15	22.7	36	22.4
45-54	23	24.2	11	16.7	34	21.1
55-64	11	11.6	11	16.7	22	13.7
≥65	12	12.6	13	19.7	25	15.6
**Total**	95	100.0	66	100.0	161	100.0

The two most common psychiatric disorders were schizophrenia (F20-F29 in ICD-10; 36.6%) and mood disorders (F30-F39; 21.1%) (Table 2). The mean duration of cumulated hospitalizations at the CHGR was 4.8 years (0–40 years ± 7.9 years). Most patients (80.7%) were prescribed psychotropic medication. The mean number of prescribed drugs was 6.7 ± 3.1.

**Table 2:** Medical, salivary, behavioural characteristics of the sample

				**n**	**%**
**ICD Code**	**ICD Label**				
F00-F09	Organic including symptomatic, mental disorders			13	8,1
F10-F19	Mental and behavioral disorders due to psychoactive substance use			20	12.4
F20-F29	Schizophrenia, schizotypal and delusionaldisorders			59	36.6
F30-F39	Mood [affective] disorders			34	21.1
F40-F49	Neurotic, stress-related and somatoform disorders			6	3.7
F60-F69	Disorders of adult personality and behavior			4	2.5
F70-F79	Mental retardation			12	7.5
F80-F89	Disorders of psychological development			6	3.7
F90-F98	Behavioural and emotional disorders with onset in childhood and adolescence			2	1.2
Unknown				5	3.1
	*n*	*%*		*n*	*%*
**Length of hospitalization**			**Plaque index**		
<1 year	90	55.9	<1	49	34.3
1-4 y	28	17.4	1-1.9	73	51.0
5-19 y	30	18.6	≥2	18	12.6
≥20 y	13	8.1	Unknown	3	2.1
**Psychotropic medication**			**Calculus index**		
Classic antipsychotic	78	48.4	<1	111	77.6
Atypical antipsychotic	77	47.8	1-1.9	22	15.4
Anxiolytics	70	43.5	≥2	7	4.9
Hypnotics an sedatives	61	37.9	Unknown	3	2.1
Antidepressants	51	31.7	**OHI-S**		
**Degree of disability**			Excellent (0)	1	0.7
Self sufficient	85	52.8	Good (0.1-1.2)	64	44.8
Partially or totally disabled	76	47.2	Fair (1.3-3.0)	59	41.3
**Saliva flow (mg/min)**			Poor (3.1-6.0)	16	11.2
≤0.3	89	60.5	Unknown	3	2.1
>0.3	58	39.5			
**Toothbrushing**					
Never	18	12.6			
< once a day	26	18.2			
Once a day	93	65.0			
Unknown	6	4.2			

One hundred and ten patients (68.3%) were free of any soft tissue lesion. The 51 remaining patients had one or several lesions: 20 patients (12.4%) had oral ulcers, 11 patients (6.8%) were diagnosed with oral candidiasis, while 8 patients (5%) exhibited oral leucoplakia.

Among the 147 patients who agreed to carry out the salivary examination, the average saliva flow rate was 0.3 g ± 0.3 g/min. Saliva flow below average (0.5 mg/min) was found in 80.3% of the patients. The buffer capacity was insufficient for 76.9% of the people, medium for 21.4% and good for only 3.6%. The number of lactobacilli was high for all patients (> 10^5^ lactobacilli/ml), the number of Streptococcus Mutans was high (> 10^5^ SM/ml) for 54.5% of the people. Finally, the caries risk was high for all the patients (CARIO-ANALYSE Microbiologic Index > 8).

Among the 143 dentate patients, the mean plaque index was 1.2 ± 0.7 (the most frequent value was 1and was found in 57.3% of the examined teeth; 0: 20.7%; 2:15.1% and 2: 6.9% and 3: 1.9%), the mean calculus index was 0.5 ± 0.6 (the most frequent value was 0 in 68.0% of the examined teeth; 1:23.9%; 2:6.4% and 3:1.7%) and the mean OHI-S was 1.7 ± 1.1. OHI-S was excellent for only 1 patient, good for 44.8% of the patients, fair for 41.3% and poor for 11.2%. Eighteen patients (12.6%) patients never cleaned their teeth.

Fifty-four patients (33.5%) declared that they don’t smoke. Twenty-four (14.9%) said they were used to smoke less than 10 cigarettes a day and 34 (21.3%) smoked 10 cigarettes a day or more.

The DMFT (Table 3) was 15.8 ± 8.8. Missing teeth represented 46.2% of the total DMFT, filled teeth, 29.7% and decayed teeth 23.4%. The mean DMFT significantly increased with age (p > 0.0001), diagnosis (ANOVA: F = 2.0225; p = 0.0475) and length of hospitalization (p = 0.0154). The DMFT distribution by type of mental disorder showed that the patients with Organic mental disorders and Mental retardation had the highest mean scores (respectively 20.8 ± 9.3 and 19.3 ± 9.0) and the patients with somatoform disorders had the lowest mean score (6.7 ± 8.3).

**Table 3:** Tooth decay index (DMFT scores) by age, gender,psychiatric disorder and length of hospitalization

	**Decayed m(sd)**	**Missing m(sd)**	**Filled m(sd)**	**DMFT m(sd)**
**Age (year)**	0.63	<0.0001*	<0.0001*	<0.0001*
<24	3.4 (4.2)	0.1 (0.5)	2.6 (2.4)	6.1 (4.7)
25-34	3.1 (2.8)	1.1 (2.4)	7.5 (5.0)	11.7 (7.2)
35-44	5.0 (6.4)	3.0 (5.0)	6.4 (5.1)	14.5 (7.8)
45-54	3.1 (2.8)	10.3 (10.3)	4.4 (5.1)	17.8 (8.2)
55-64	4.4 (4.9)	10.1 (8.1)	4.5 (4.5)	19.0 (7.1)
65-74	4.2 (3.6)	15.4 (8.6)	3.0 (4.3)	22.6 (8.2)
75-90	1.7 (2.4)	21.6 (9.1)	0.8 (2.5)	24.1 (6.1)
**Gender**	0.20	0.68	0.55	0.512
Female	3.1 (3.7)	7.8 (9.8)	4.5 (4.6)	15.4 (9.1)
Male	4.1 (4.8)	7.0 (9.2)	4.9 (5.0)	16.1 (8.6)
**Disease**	0.38	0.0005*	0.16	0.0475*
Organic mental disorders	2.6 (3.2)	17.5 (10.6)	0.7 (1.9)	20.8 (9.3)
Disorders due to psychoactive substance	5.1 (3.1)	6.6 (8.5)	5.0 (4.7)	16.7 (7.4)
Schizophrenia	3.7 (4.6)	5.5 (9.0)	5.5 (5.1)	14.6 (8.7)
Mood disorders	2.5 (2.6)	6.8 (8.3)	6.2 (4.8)	15.5 (8.1)
Somatoform disorders	3.5 (4.3)	1.3 (2.4)	1.8 (2.6)	6.7 (8.3)
Disorders of adult personality	4.0 (3.6)	1.5 (1.9)	8.5 (4.0)	14.0 (6.8)
Mental retardation	4.2 (5.4)	11.2 (9.8)	4.0 (5.4)	19.3 (9.0)
Developmental disorders	5.7 (9.6)	9.3 (10.1)	0.7 (1.6)	15.7 (12.8)
Behavioral and emotional disorders	8.5 (10.6)	0.0 (0.0)	3.5 (0.7)	12.0 (9.9)
**Length of hospitalization (year)**	0.41	**<0.0001**	**0.0049**	**0.0154**
<1	3.7 (4.0)	6.4 (9.1)	5.0 (4.9)	15.1 (8.6)
1-4	3.4 (3.2)	4.6 (7.1)	6.1 (5.5)	14.1 (8.2)
5-19	4.9 (6.4)	7.7 (9.1)	4.53 (4.61)	17.1 (8.6)
≥20	2.2 (2.6)	19.6 (9.2)	0.8 (1.2)	22.6 (8.2)
Total	3.7 (4.4)	7.3 (9.4)	4.7 (4.9)	15.8 (8.8)

NS = Not Significant p > 0.05.

*ANOVA, p < 0.05.

Out of the 143 dentate patients, 23.0% were caries free, 34.8% had 1 to 3 decayed teeth and 42.2% had more than 4 decayed teeth.

Among the 161 examined patients’ teeth altogether, 13.31% of the teeth were decayed, 17.1% had been filled, 26.4% had been lost because of caries (31.5% lost because of caries or other reasons).

Bivariate analysis using Chi-square tests (Table 4) showed that DMFT over 16 was associated with age over 45 (p < 0.0001), length of hospitalization over 5 years (p = 0.0233), disability (p = 0.0021) and plaque index over 1 (p = 0.0111). The subjects were divided into two categories according to the DMFT (DMFT-16): 16 or less (n = 80) and over 16 (n = 81). The cutoff point (16.0) was the mean DMFT (15.8 ± 8.8).

**Table 4:** Bivariate analysis of the number (%) of psychiatric patients for the DMFT-16 (less or more than 16)

		**DMFT-16**		**χ**^***2***^
	**n**	**≤16 n(%)**	**>16 n(%)**	**p value**
**Age**				
≤45	80	55 (68.8)	25 (30.9)	23.1
>45	81	25 (31.3)	56 (69.1)	*p < 0.0001
**Gender**				
Men	95	48 (60.0)	47(58.0)	0.06
Women	66	32 (40.0)	34 (42.0)	p = 0.7989
**Psychiatric diagnosis**				
Disorders due to psychoactive substance	20	9 (15.0)	11 (20.8)	1.1
Schizophrenia	59	34 (56.7)	25 (47.2)	p = 0.5645
Mood disorders	34	17 (28.3)	17 (32.1)	
**Length of hospitalization (year)**				
≤5	118	65 (81.3)	53 (65.4)	5.1
>5	43	15 (18.8)	28 (34.6)	*p = 0.0233
**Disability**				
Self sufficient	85	52 (65.0)	33 (47.7)	9.5
Partially or totally disabled	76	28 (35.0)	48 (59.3)	*p = 0.0021
**Saliva flow (g/min)**				
≤0.3	89	50 (64.9)	39 (55.7)	1.3
>0.3	58	27 (35.1)	31 (44.3)	p = 0.2533
**Plaque index**				
≤1	81	50 (66.7)	31 (45.6)	6.5
>1	62	25 (33.3)	37 (54.4)	*p = 0.0111

*Significant difference between two groups by χ^2^ test.

Logistic regression models were used to identify factors associated with the DMFT-16. All the variables listed in Table 3 and Table 4 were introduced in the first model in order to control possible confounding effects. In SPSS, the binary logistic regression according to the “forward LR” method kept 2 variables. The logistic regressions analysis showed that age and Degree of disability were significant contributors to the fact of having a high DMFT. Age and degree of disability explained 24.5% (R2) of the DMFT-16’s variations.

Among the 143 dentate patients, 122 (93.0%) needed dental care: 41 (28.7%) needed extractions (3.1 ±3.00 teeth extractions per people), 114 (79.7%) needed restorative care (4.0 ± 3.2 dental cares per person) and 31(21.7%) needed crowns (1.7 ± 1.10 crown per patient).

Removable dentures were needed by 66 patients (41.0%) in order to replace one or several missing teeth (some patients needed several dentures): 15 (9.3%) needed one or two simple dentures, 44 (27.3%) needed one or two partial dentures, 11 (6.8%) needed one or two complete dentures, 6 (3.7%) had a denture which needed to be repaired.

Eighteen patients (11.2%) were totally edentulous and 10 (6.2%) had one edentulous arch. Among the 18 (11.2%) totally edentulous patients, 10 (55.6%) had a full set of complete dentures, 3 (16.8%) wore only an upper complete denture, 5 (27.8%) had no denture at all.

The 120 (83.9%) partially or totally edentulous patients had 7.0 ± 8.3 (from 0 to 28) missing (and not replaced) teeth. (Here, in order to evaluate the prosthetic needs, a missing tooth was considered as not replaced if there was still enough space to allow its replacement by a prosthetic tooth. Extracted teeth for orthodontic reasons were not considered as missing not replaced teeth).

## Discussion

In summary, the main results of this study were the DMFT (15.8) of psychiatric inpatients and especially the mean number of missing (7.3) and decayed teeth (3.7). Eleven point two percent was totally edentulous. The dental unmet treatment needs were important: 93% of the patients needed dental care and 83.9% needed teeth replacement. Saliva flow was extensively below normal and oral hygiene was poor (Plaque index = 1.2 meaning between one third and two third of the teeth surfaces covered by soft debris). Bad dental health was associated with age and the degree of disability.

Regarding the validity of our results, 24 (13%) people were excluded because they either refused to participate (n = 14) or were unable to cooperate (n = 10). Thus, the response rate was 87%. This was a good response rate compared to similar studies where the average response rate was 82% [7,9-12,16,18,27] (from 58% [10] to 97% [13]). But this might introduce a selection bias. Indeed, regarding people unable to cooperate (n = 10), it is difficult to brush their teeth regularly. Thus, oral health of the sample might have been worse if they had been included. Regarding people who refused to participate, we can make no assumption.

Some information was asked to the patient (tooth brushing and smoking habits). Their reliability and validity is questionable. For each of these questions, the nurse was also questioned. When we had any doubts about the patient’s answer, we did not record it.

Compared to literature (with mean age = 47.3 ± 5 years), the mean DMFT (15.8) was one of the lowest for non-fluoride water areas. It was lower than that found in middle-income countries: Serbia (DMFT = 24.4 with mean patients’ age = 46) [12] and Turkey (DMFT = 19,25 with mean age = 52,3) [16], and high-income countries like Israel (DMFT = 23.8 with mean age = 53) [11]. It was slightly higher than that found in Taiwan (13.9 with mean age = 51) [13].

Missing teeth (46.2%) accounted for the largest portion of the number of teeth ever affected by tooth decay (DMFT) as ever in literature. The number of filled teeth may be a reflection of the available treatments for the patients. In our study, proportion of filled teeth accounted for 29.7% of the DMFT. It was the highest in literature. In our population, dental treatment was available and free. On the opposite, in a lot of studies, no routine treatment was available. Only emergency cares in the form of extractions were provided.

The number of decayed teeth (3.7) was still high. Preventive actions are still needed in order to reduce the incidence of tooth decay.

Regarding edentulousness, the proportion of totally edentulous in-patients (11.2% with mean age = 47) was lower than that found in similar studies (mean ages ranges 42–52), where rates of edentulousness ranged from 18% to 26% [11,12,16] except in one study in Hong Kong: 7% (mean age = 45) [9].

Compared to low income French population (DMFT = 14.8 in the 35–44 year-olds and 24.5 in the 65–74 year-olds) [28,29], the DMFT (14.5 in the 35–44 year-olds and 22.6 in the 65–74 year-olds) was about the same (Table 5). But the number of decayed teeth was almost four times higher (5.0 in the 35–44 and 4.2 in the 65–74) and the number of missing teeth (because of tooth decay) was lower (3.0 in the 35–44 and 15.4 in the 65–74). The number of filled teeth (6.4 in the 35–44 and 3.0 in the 65–74) was almost 1.5 times lower.

**Table 5:** Comparison with French population

	**DMFT**	**D**	**M**	**F**
**Our study**				
35–44	14.5	5.0	3.0	6.4
65-74	22.6	4.2	15.4	3.0
**French population (1995)**				
**35–44** means	14.6	1.2	3.0	10.4
High occupational group (OG)	13.7	0.9	2.0	10.8
Low OG	14.8	1.3	3.6	9.9
**65-74** means	23.3	1.1	16.9	5.2
High OG	19.4	0.9	10.2	8.4
Low OG	24.5	1.2	19.4	4.0

Thus, the psychiatric patients had considerably more important treatment needs than the general population (four times more untreated decayed teeth). Even if the psychiatric hospital had a dental service, there was still a lack of dental care compared to the French population. The dental office didn’t manage to cope with the extensive treatment needs.

Actually, the gap between psychiatric in-patients and French population (not only low-income) might probably be higher. Available data regarding oral health of French population are old (1997 and 1999). As in many industrialized countries, oral health of French population might have improved over time.

This article aimed at showing to psychiatrists the oral health and needs of psychiatric patients. Tooth decay and tooth loss in psychiatric patients are avoidable. But the dental team, when existing, really needs psychiatrist’s support to change things. Psychiatrists and their care-giving staff must be aware and get involved in dental issues. Considering the findings of this study, the following suggestions are made:

– As the dental care needs are often unmet, as prevalence of oral lesions and caries’ risk are high, psychiatrists must send patients to frequent and systematized clinical dental visits for screening and treatment of early caries and mucosa lesions and for professional fluoride varnish applications.

– As bad oral health is associated with invalidity, a special attention shall be put to oral care as soon as patients are not able to get dressed on their own or to wash themselves.

– As saliva flow is often below normal, they can prescribe artificial salivary products to combat xerostomia.

– As saliva buffer capacity needs to be improved, sodium bicarbonate mouthwash may be used every day.

– Additionally, the number of Lactobacilli needs to be reduced. An antiseptic mouthwash (chorhexidine) may be used for short periods alternatively with the fluoride mouth rinse.

– As oral hygiene is usually poor, chemical adjuncts may be needed for long term plaque control. Oral rinses containing fluorides could be provided as they can be used for a long time without modifying the balance of the oral flora (unlike chlorhexidine).

– Dental hygienists or trained nurses are needed for daily oral hygiene: every patient needs to be checked that he has a toothbrush and toothpaste. He should be given some if needed. The primary preventive effort has to focus on tooth brushing: educational instruction and motivation for able people and supportive instruction for nurses to provide or assist dependent patients in their daily oral hygiene. Mechanical toothbrush has proved to be effective for psychiatric patients [30].

## Conclusions

This study highlighted that the DMFT and the number of missing teeth were lower than those found in most other countries; the number of filled teeth was higher. Dental care was accessible and free.

But the need of care of psychiatric patients still exceeded that of the general population. We know now that tooth decay is an avoidable disease. Thus, preventive measures have to be implemented to lower the incidence of tooth decay and alleviate the burden of untreated decays. Screening must be systematized and access to care must be encouraged.

Psychiatrists, physicians, nurses, caregivers, hospital administrators, must be aware of poor dental health of mentally ill people and coordinate their efforts to facilitate prevention and access to dental care.

This study was partly funded by the CHU of Rennes and the Faculty of Dentistry of Rennes.

## Competing interests

The authors declare that they have no competing interests.

## Authors’ contributions

VB conceived the study, performed its design, the acquisition of data, the statistical analysis and the drafted the manuscript. VK has made substantial contribution in the interpretation of data and in the critical manuscript revision for important intellectual content. CL has made substantial contribution in the design and coordination of the study and in the interpretation of data. GT has made substantial contribution in the interpretation of data and in the critical manuscript revision for important intellectual content. FR performed the design of the study, has made substantial contribution in the acquisition of data and in the interpretation of data. All authors read and approved the final manuscript.

## Pre-publication history

The pre-publication history for this paper can be accessed here:

http://www.biomedcentral.com/1471-244X/13/227/prepub
